# Valproic Acid and Lamotrigine Differentially Modulate the Telomere Length in Epilepsy Patients

**DOI:** 10.3390/jcm14010255

**Published:** 2025-01-03

**Authors:** Salvador Sánchez-Badajos, Alberto Ortega-Vázquez, Marisol López-López, Nancy Monroy-Jaramillo

**Affiliations:** 1 Doctorado en Ciencias Biológicas y de la Salud, Universidad Autónoma Metropolitana, Mexico City 04960, Mexico; 2233800589@alumnos.xoc.uam.mx; 2 Departamento de Sistemas Biológicos, Universidad Autónoma Metropolitana Unidad Xochimilco, Mexico City 04960, Mexico; aortega@correo.xoc.uam.mx (A.O.-V.); mlopez@correo.xoc.uam.mx (M.L.-L.); 3 Departamento de Genética, Instituto Nacional de Neurología y Neurocirugía Manuel Velasco Suárez (INNNMVS), Mexico City 14269, Mexico

**Keywords:** epilepsy, telomere length, mtDNA copy number, antiseizure drugs, biological aging

## Abstract

**Background/Objectives**: Antiseizure drugs (ASDs) are the primary therapy for epilepsy, and the choice varies according to seizure type. Epilepsy patients experience chronic mitochondrial oxidative stress and increased levels of pro-inflammatory mediators, recognizable hallmarks of biological aging; however, few studies have explored aging markers in epilepsy. Herein, we addressed for the first time the impact of ASDs on molecular aging by measuring the telomere length (TL) and mtDNA copy number (mtDNA-CN). **Methods**: We used real-time quantitative PCR (QPCR) in epilepsy patients compared to matched healthy controls (CTs) and assessed the association with plasma levels of ASDs and other clinical variables. The sample comprised 64 epilepsy patients and 64 CTs. Patients were grouped based on monotherapy with lamotrigine (LTG) or valproic acid (VPA), and those treated with a combination therapy (LTG + VPA). Multivariable logistic regression was applied to analyze the obtained data. **Results**: mtDNA-CN was similar between patients and controls, and none of the comparisons were significant for this marker. TL was shorter in not seizure-free patients than in CTs (1.50 ± 0.35 vs. 1.68 ± 0.34; *p* < 0.05), regardless of the ASD therapy. These patients exhibited the highest proportion of adverse drug reactions. TL was longer in patients on VPA monotherapy, followed by patients on LTG monotherapy and patients on an LTG + VPA combined scheme (1.77 ± 0.24; 1.50 ± 0.32; 1.36 ± 0.37, respectively; *p* < 0.05), suggesting that ASD treatment differentially modulates TL. **Conclusions**: Our findings suggest that clinicians could consider TL measurements to decide the best ASD treatment option (VPA and/or LTG) to help predict ASD responses in epilepsy patients.

## 1. Introduction

Epilepsy is one of the most common neurological diseases worldwide, affecting around 50 million people of all ages around the world. The prevalence of epilepsy varies between countries, and nearly 80% of people with epilepsy live in low- and middle-income countries [1]. For the vast majority of people with epilepsy, the initial therapy consists of pharmacological treatment, where there is a wide variety of antiseizure drugs (ASDs) [2], and the choice of them varies according to the different types of seizures and epileptic syndromes [3]. Epilepsy is the leading cause of outpatient consultation at the Manuel Velasco Suárez National Institute of Neurology and Neurosurgery (INNNMVS), a third-level hospital in Mexico City, where valproic acid (VPA) and lamotrigine (LTG) are two of the most widely used ASDs [4].

Telomeres are repetitive noncoding DNA sequences that protect the ends of chromosomes and provide genomic stability. The telomere length (TL) decreases with each cell division due to incomplete replication of linear chromosomes [5,6] and has been suggested as a “mitotic clock” indicating cell age [7]. There are reports supporting a relationship between some medicines that are used in the management of cardiovascular diseases, diabetes, and menopause and their positive protective effects against TL shrinking [8]. Similarly, some psychotropic medications may modulate TL; for instance, clinical evidence suggests that lithium may attenuate telomere shortening in patients with bipolar disorder (BD) [9,10,11,12]. The length of the leukocyte TL has also been associated with the response to some antidepressant drugs [13,14]. Antipsychotics could prolong or retain TL in peripheral blood mononuclear cells [15] and might influence telomerase activity [16]. In this regard, some studies have found no effect of VPA, LTG, carbamazepine, or any combination of them on TL in BD patients [10,17], while VPA, in a rat model for autism, induced an increased gene expression of telomerase in vivo and in vitro [18]. There is only one observational study that has shown telomere shortening in drug-resistant epilepsy patients [19].

Evidence indicates the existence of a strong link between TL shortening and metabolic and mitochondrial compromise, which are central mechanisms to cells’ functional decline during aging [20,21,22]. Telomere shortening may affect mitochondria activity through nuclear signaling to mitochondria [21], inducing alterations in the electron transport chain, which will result in an increased generation of reactive oxygen species (ROS), reduced ATP levels, and more DNA damage being induced. In turn, the oxidative stress will cause greater telomere shrinkage [23]. Indeed, a Mendelian randomization (MR) analysis found a positive causal effect of TL on mtDNA-CN, suggesting a complex interrelationship between these two markers in the aging process [22].

Mitochondrial dysfunction has been implicated in the pathophysiology of neurological and psychiatric disorders, and, particularly in epilepsy, it has been identified as one potential cause of epileptic seizures [23]. On the other hand, a relation between psychotropic medication and the mtDNA copy number (mtDNA-CN), a measure of the number of mitochondrial genomes per cell, has also been postulated. It is known that antipsychotics and mood stabilizers (including VPA and LTG) may affect the function of mitochondria in BD [12,24,25,26]. Antipsychotics may have an effect in reducing mtDNA-CN in patients with major depression disorder [27] or with psychosis [28]. In contrast, the VPA concentration has been shown to be positively correlated with peripheral mtDNA-CN and better cognitive performance in BD patients, distinguishing responders vs. non-responders to that ASD [29].

TL and mtDNA-CN are markers of biological aging that have been studied in several neurological disorders [19,28,29,30,31,32,33], reporting differences when compared with healthy controls. The accumulation of adverse effects of some conventional ASDs over time, as well as pro-inflammatory and oxidative stress conditions produced by epileptic seizures, may contribute to biological aging in patients with epilepsy [19,34]. To the best of our knowledge, no report has examined the effect of ASD treatment on TL and mtDNA-CN in patients with epilepsy. Herein, we investigated whether ASDs modulate biological aging by comparing TL and CN-mtDNA in patients with epilepsy to those of age- and sex-matched healthy controls.

## 2. Materials and Methods

### 2.1. Study Participants

Sixty-four unrelated patients with epilepsy (18–72 years old; thirty-three females) were consecutively recruited from the Epilepsy Clinic at the INNNMVS. Sixty-four clinically healthy controls were matched by age and sex. Controls enrolled in the study were college students or unrelated companions of patients, with no family history of epilepsy or any neurological disease, and who were not taking any ASD. All participants were Mexican Mestizos (MM), with at least the two previous generations born in Mexico. This study was carried out in accordance with the latest version of the Declaration of Helsinki, and the study design was reviewed and approved by the local Research and Ethics Committees (registration numbers: INNNMVS_38/19 and UAM-X #34605034). Written informed consent was obtained from all participants after the nature of the procedures had been fully explained.

### 2.2. Clinical Data of Patients

A diagnosis of epilepsy was established for each patient by an expert neurologist in this study based on international criteria [35,36]. All patients were under pharmacological treatment and categorized into three groups, including monotherapy (LTG or VPA) and combined therapy (LTG + VPA), as follows: with LTG (*n* = 18), with VPA (*n* = 19), and 27 patients with combined therapy. Patients were taking the mentioned ASD treatment at least six months before the study. Data regarding dose and adjusted plasma concentrations of ASD, number of epileptic crises per year, and type of seizure (focal vs. generalized) were extracted from their clinical records. In compliance with the operational definition of seizure freedom of the International League Against Epilepsy (ILAE), patients were considered “seizure-free” following an intervention after a period without seizures equal to three times the longest pre-intervention inter-seizure interval over the previous year had elapsed [37]. Adverse drug reactions (ADRs) to ASDs were personally queried with a questionnaire ad hoc. ADRs were classified into general, gastrointestinal, cutaneous, neurological, and psychiatric reactions for all patients, and patients were categorized by ASD (Table S1).

### 2.3. Relative Quantification of Telomere Length and mtDNA Copy Number

Peripheral blood samples (12 mL) were collected from all subjects, and genomic DNA was isolated by standard procedures. The relative quantification of the leukocyte TL [38] and mtDNA-CN [39] were assessed by real-time quantitative PCR (QPCR) as previously described. For both quantifications, a standard curve of serial dilutions of a commercial DNA from CEPH individual 1347–02 was included in each run (Thermofisher, Écublens, Switzerland). A relative measure of TL was calculated as a telomere hexanucleotide repeat/single copy gene (T/S) ratio and as a mtDNA/nuclear DNA (*ND3*/*TH*) ratio. All PCRs were performed using KAPA SYBR Fast ABI Prism qPCR Master Mix on a QuantStudio™ 5 Real-Time PCR System (Applied Biosystems, Waltham, MA, USA). All DNA samples were run in four replicates on separate plates for each sequence of interest, but in the same well positions. The inter-assay and intra-assay coefficients of variation were calculated and accepted when <10%.

### 2.4. Statistical Analyses

Statistical analyses were performed using R version 4.4.2 (R Core team, Vienna, Austria) [40,41]. The normality of the data was estimated with Kolmogorov–Smirnov and Shapiro–Wilk tests, and then, mean values were compared between groups using Student’s t test, U Mann–Whitney test, or Kruskal–Wallis test, accordingly. Correlation analyses were carried out using simple linear correlation approaches. Subsequently, for the patient data, a multivariate regression analysis was used. Covariates included in the analyses were age and sex for all participants, while seizure freedom, dose, and plasma concentrations (PCs) of each ASD were only included for patients. A *p*-value < 0.05 was considered statistically significant in all analyses.

## 3. Results

The socio-demographic characteristics of the 128 participants (64 patients with epilepsy and 64 controls) are presented in Table 1. Both groups under study included 31 males and 33 females of comparable ages.

All patients were being treated with ASDs, and 34.4% of them were seizure-free. According to the ASD treatment received, 28%, 53%, and 26% were seizure-free patients on LTG, VPA, or combined therapy, respectively. The seizure types were 66.13% focal onset and 33.87% generalized onset. The most frequent ADRs were neurological and psychiatric (memory failure and aggression/irritability, respectively). Then, when patients were grouped by ASD, the most frequent ADRs by group were headache in LTG monotherapy; mood changes in VPA monotherapy; and nervousness or distress in the group receiving combined therapy (Table S1). The adjusted plasma ASD concentrations were 1.5 ± 1.5, 4.4 ± 1.3, and 3.7 ± 1.8/2.7 ± 1.6 µg/mL/dose/Kg for LTG, VPA, and combined therapy (LTG + VPA), respectively (Table 1). Seven patients on LTG monotherapy were found to have subtherapeutic PC (<3 µg mL^−1^) and eleven were within therapeutic levels (3–15 µg mL^−1^) [42]. All patients on VPA monotherapy had levels that fell within the therapeutic range (8.9–115 µg mL^−1^) [42,43]. Regarding the group receiving combined therapy, most of the patients were found to be within the therapeutic levels for LTG (21/26, 81%), one had subtherapeutic levels, and five were at supratherapeutic levels. The therapeutic range for VPA was found to be within therapeutic levels for all but one patient, who was at supratherapeutic levels (>115 µg mL^−1^).

**Figure 1:**
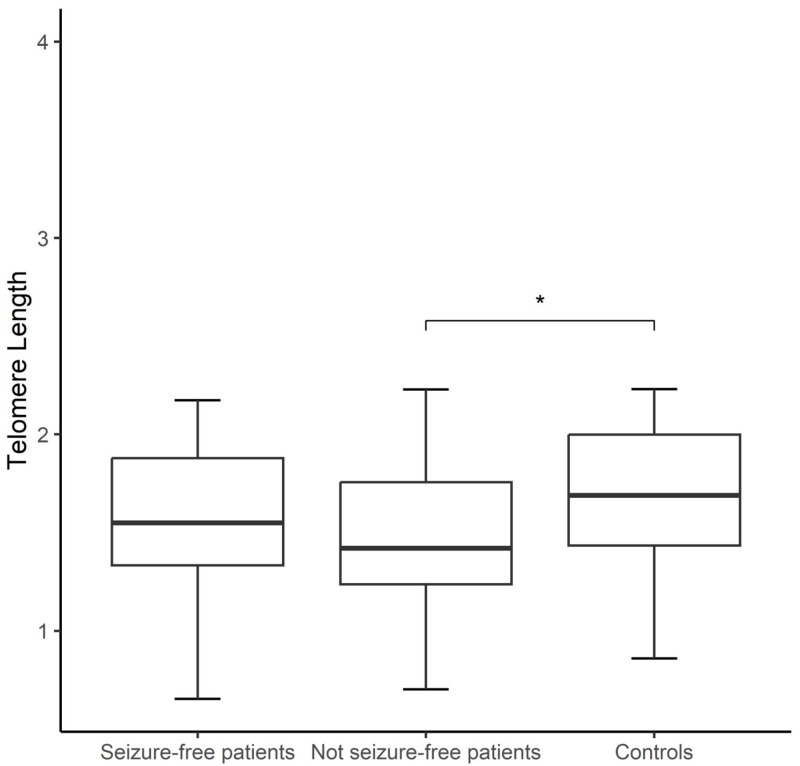
Boxplot showing comparison of telomere lengths (TLs) of seizure-free patients (*n* = 22), not seizure-free patients (*n* = 42), and controls (*n* = 64). Student’s *t*-test was used. Significance is denoted by an asterisk as follows: * *p* < 0.05.

The epilepsy patients presented shorter telomeres when compared to controls; however, this difference was only significant between not seizure-free patients, regardless of the ASD treatment, and controls (1.50 ± 0.35 vs. 1.68 ± 0.34; *p* < 0.01; Figure 1). Among the controls, women exhibited shorter TLs than men (1.09 ± 0.22 vs. 1.04 ± 0.22; *p* = 0.04), but this was not observed in patients. After comparing patients categorized by seizure type (focal vs. generalized, groups of similar age, *p* > 0.05) to controls, the group of patients with focal seizures exhibited shorter TLs compared to controls (1.48 ± 0.36 vs. 1.68 ± 0.34; *p* < 0.01); however, no difference was observed between patients with focal and generalized seizures. mtDNA-CN was similar between patients and controls (0.41 ± 0.11 vs. 0.42 ± 0.10; *p* = 0.07), and none of the above comparisons (seizure type and seizure freedom) were significant for this marker.

**Figure 2:**
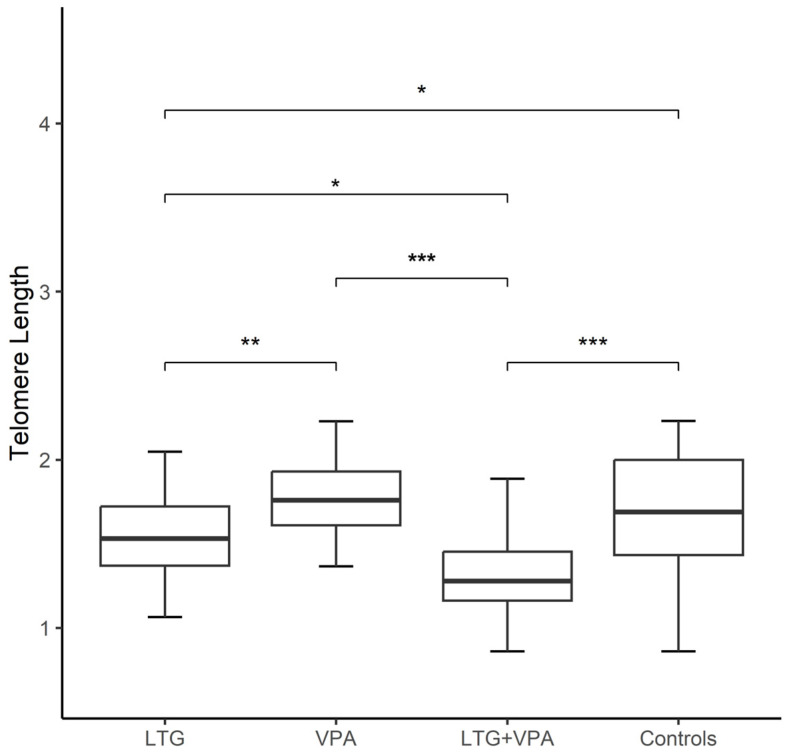
Boxplot showing comparison of telomere lengths (TLs) among epilepsy patients grouped by antiseizure drug treatment (ASD) and controls (*n* = 64) as follows: patients on lamotrigine (LTG, *n* = 18); patients on valproic acid (VPA, *n* = 19); patients on LTG + VPA (*n* = 27). TL of each group of epilepsy patients compared to controls and TL differences among patients grouped by ASD treatment was performed with Kruskal–Wallis test. Significance is denoted by asterisks as follows: * *p* < 0.05, ** *p* < 0.01, and *** *p* < 0.001.

Both aging markers were compared between controls and patients categorized based on their ASD treatment: LTG (18), VPA (19), and LTG + VPA (27). The Kruskal–Wallis test revealed TL differences among patients grouped by ASD treatment (*p* < 0.05) according to the following ranking: TL of patients on LTG + VPA < TL of patients on LTG < TL of patients on VPA (1.36 ± 0.37 vs. 1.50 ± 0.32 vs. 1.77 ± 0.24, respectively); in contrast, the controls exhibited a mean value of TL of 1.68 ± 0.34. Using the Mann–Whitney U test, differences were observed between the TLs of patients receiving a combined ASD therapy vs. controls (1.36 ± 0.37 vs. 1.68 ± 0.34; *p* < 0.001), as well as the TLs of patients on LTG vs. controls (1.50 ± 0.32 vs. 1.68 ± 0.34; *p* < 0.05) (Figure 2). In contrast, the mtDNA-CN of controls vs. patients categorized by ASD treatment did not show differences (Kruskal–Wallis test, 0.42 ± 0.10 vs. 0.45 ± 0.12, 0.40 ± 0.09, and 0.39 ± 0.10 for patients on VPA, LTG, and LTG + VPA, respectively).

**Figure 3:**
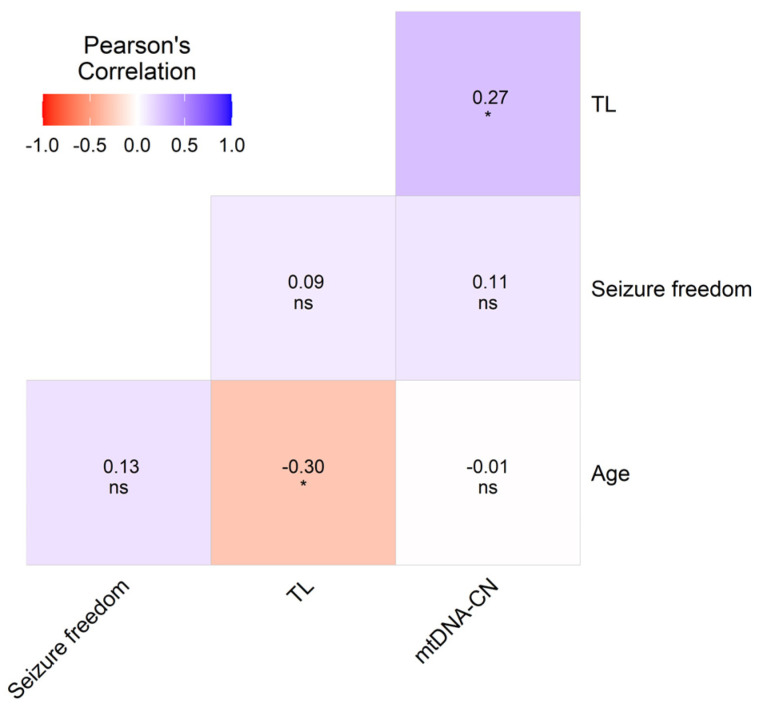
Heatmap matrix depicting the multiple Pearson correlations between TL, mtDNA-CN, sex, age, and clinical variables with significant values in included epilepsy patients (*n* = 64). Significance is denoted by asterisk as follows: * *p* < 0.05.

The correlation analysis between the TL and chronological age (in years) in both groups of patients studied showed a moderate negative correlation: R = −0.30 and *p* = 0.02 in patients and R = −0.39 and *p* < 0.01 in controls. In addition, the correlation of TL vs. mtDNA-CN was significant in patients with epilepsy (R = 0.27; *p* = 0.03) (Figure 3), and the analysis divided by ASD treatment revealed that this observation only persisted in the group receiving LTG + VPA therapy.

**Figure 4:**
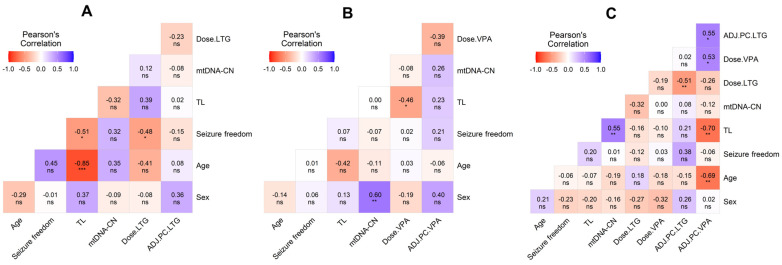
Heatmap matrix depicting the multiple Pearson correlations between TL, mtDNA-CN, sex, age, and clinical variables with significant values. (**A**) Heatmap depicting the correlation in epilepsy patients on LTG. (**B**) Heatmap depicting the correlation in epilepsy patients on VPA. (**C**) Heatmap depicting the correlation in epilepsy patients on LTG + VPA. Significance is denoted by asterisks as follows: * *p* < 0.05, ** *p* < 0.01., and *** *p* < 0.001. ADJ.PC: adjusted plasma concentration of antiseizure drug.

We performed multiple logistic regressions (MLRs) adjusting for confounding factors in all patients (Figure 3) and the patients grouped by ASD treatment (Figure 4). The TL was considered the independent variable, whereas ASD treatment, sex, age, seizure freedom, ASD dose, adjusted plasma concentrations (ADJ PCs), ADRs, and mtDNA-CN were dependent variables. The *p*-values obtained with this analysis were only significant for TL vs. age (R = −0.30, *p* < 0.05), and the TL correlated with mtDNA-CN (R = 0.27, *p* < 0.05) (Figure 3). The correlation analysis between types of ASD adverse drug reactions and the aging markers in epilepsy patients did not show any significant results (Table S2).

We then performed an MLR analysis on patients based on their ASD therapy, which included doses and adjusted plasma concentrations. The analysis of LTG monotherapy showed strong negative correlations between the TL and seizure freedom (R = −0.51; *p* < 0.05) and LTG dose and seizure freedom (R = −0.48; *p* < 0.05) (Figure 4A). The output for this analysis in patients on VPA monotherapy revealed a strong positive correlation between mtDNA-CN and sex (women had a higher copy number) (R = 0.60; *p* < 0.01) and a significant negative relation between VPA dose and TL (R = −0.46; *p* < 0.05) (Figure 4B). The MLR analysis in the group of patients on LTG + VPA also showed a strong correlation between both studied aging markers (mtDNA-CN vs. TL, R = 0.55; *p* < 0.01), while the adjusted plasma concentrations of VPA were negatively correlated with mtDNA-CN (R = −0.40; *p* < 0.05) and with TL (R = −0.68; *p* < 0.01) (Figure 4C).

## 4. Discussion

Mortality rates are 2–3 times higher in epilepsy patients than in the general population. The average reduction in life expectancy in these patients is 10.91–11.84 years (i.e., symptomatic cases) compared to the general population [44]. On the one hand, telomere shortening and mtDNA-CN are associated with human diseases and reduced life expectancy. On the other hand, seizures, accidents, and psychological factors that are common in epilepsy may, in turn, be associated with changes in the TL and mtDNA-CN, and perhaps with other aging markers. The observed reduction in life expectancy in these patients could be linked to the concept of accelerated cellular aging and be differentially modulated by ASDs. Some reports suggest potential aging-modulating properties for VPA and LTG: (i) increasing the lifespan in experimental models [45,46], and (ii) VPA decelerating epigenetic aging, which was found in peripheral samples of BD patients; however, further investigation is required [47].

This is the first study in which two aging markers are simultaneously evaluated in controls and patients with epilepsy under different ASD treatments: monotherapy (LTG or VPA) or in a combination scheme (LTG + VPA). Our results demonstrated shorter TLs in epilepsy patients compared with controls, but far from associating this marker with an increased risk of the disease, we highlight the potential differential aging-modulating properties of these ASDs, which might be dose-dependent.

After categorizing patients by seizure freedom and type, shorter TLs were shown in not seizure-free patients (i.e., “non-responders”, regardless of the ASD treatment) and in those with focal seizures. This agrees with a previous work, where the authors compared the TLs of patients and controls and observed significantly shorter TLs in the group of drug-resistant epilepsy patients [19].

After comparing patients categorized by ASD therapy, significant differences in TLs among them persisted, particularly for patients on LTG monotherapy and combination therapy, suggesting a different modulation of the TL depending on the ASD studied. Of note, these two groups included a high percentage of not seizure-free patients (72% and 74%, respectively). In particular, the LTG monotherapy group showed a negative correlation between LTG dose and seizure freedom and between TL and seizure freedom. This last finding is in accordance with that reported in animal studies showing that a shortened TL increased the seizure frequency [16,48].

Beyond the therapeutic effects as ASDs, VPA and LTG exhibit potential effects as mood stabilizers. In this regard, there are previous reports of their use for BD and its relationship with the TL and mtDNA-CN. Some of these suggest that LTG [49] and VPA [48] may protect against oxidative stress and possibly TL shortening, while others found no differences in TLs between ASD-treated BD patients and controls [10]. However, there are no reports in epilepsy patients of the impact of LTG and VPA treatment on the TL and/or mtDNA-CN. This work demonstrated that epilepsy patients who are treated with LTG or LTG + VPA therapy had shorter TLs than patients on VPA monotherapy and controls. Also, the MLR analysis only showed a positive correlation between both studied aging markers in the group of patients on LTG + VPA. A similar correlation has been previously documented, mainly in old patients with cancer [50] and in patients with Parkinson’s disease [51]. The three groups of patients on different ASD treatments did not show differences in age or sex among them, ruling these out as variables associated with this correlation. The strong correlation observed might be a compensation for the insufficient cellular energy supply due to the mitochondrial dysfunction in epilepsy patients on polytherapy, whereby increasing mtDNA-CN occurs to maintain normal mitochondrial functions [51].

Regarding the TL results observed in patients who were treated with VPA monotherapy, its neuroprotective properties could be involved [52]. VPA functions as an inhibitor of histone deacetylases (HDACi), activating the transcription from many promoters [53]; also, it has been reported that the neural progenitor cells of rats exposed to VPA in vitro exhibited increased telomerase expression [18,54]. Nonetheless, we also observed that a higher VPA dose on monotherapy, a shorter TL (Figure 4B), and plasma VPA concentrations negatively correlated with the TL and mtDNA-CN in the combined scheme (Figure 4C). This apparent contradiction of VPA treatment warrants deeper investigation. Individual variability in VPA metabolism could also play a significant role in drug clearance; therefore, future research should also address the pharmacogenomic aspects of VPA to better understand the specific impacts of this drug on the TL and mitochondrial function.

On the other hand, the VPA concentration has been shown to be positively correlated with mtDNA-CN, better cognitive performance, and a response to VPA in BD patients [55]. We did not evaluate cognition, but the VPA concentrations were at therapeutic levels, and more than half of the individuals in the VPA monotherapy group were seizure-free patients. The TL is modulated by a plethora of intrinsic and extrinsic factors, including the effect of pharmacologically active substances. Indeed, a recent review proposes that integrating TL measurements into personalized medicine procedures could aid significantly in individualizing the treatment plan [8].

The synergistic scheme of LTG and VPA is an effective treatment of refractory epilepsy in children and adults and has been considered the best combination therapy in a retrospective study [56]. However, VPA can decrease LTG clearance by 54% in combination therapy [57]. In this sense, the patients with the combined therapy showed the highest percentage of ADRs (Table S1), and somehow, their accumulation over time, as well as the risks factors mentioned earlier herein, could jointly accelerate biological aging in patients with epilepsy. Another study also found that female patients had higher oxidative stress levels than controls, and that this was more pronounced in patients on ASD polytherapy vs. those who were on monotherapy [58] Therefore, we cannot rule out that higher ASD doses or the combined ASD therapy may lead to shortening of telomeres in patients with epilepsy, as was observed in the present study.

Little is known about ASDs’ interference with mitochondria; e.g., in vitro, the mtDNA-CN increased after VPA treatment in a dose-dependent manner [59,60]. Conversely, it has been documented that LTG has toxic effects on mitochondria [61]. On the one hand, ASDs are known to induce epigenetic modifications with unknown consequences, while on the other hand, VPA, LTG, and other ASDs are known to exert HDACi properties [62,63]. For instance, LTG monotherapy led to lower serum folate levels in patients with epilepsy, which in turn may affect the DNA methylation process [64]. Some differences in epigenetic age acceleration in BD patients taking combinations of mood stabilizers (including VPA) vs. those taking no medication/monotherapy have been reported, as well [47]. The comparison of TLs between patients and controls, and the positive correlation of TL with mtDNA-CN that was exclusively found in patients on combined therapy (LTG + VPA), indicates a mechanism related to biological aging participating in the physiopathology of epilepsy, where one of the potential implicated factors might be oxidative stress. In fact, the marked oxidative stress in epilepsy may accelerate the telomere erosion observed, which has been previously shown in epilepsy patients [19,65].

Contrary to the TL, mtDNA-CN was similar between the controls and patients, as well as among patients, regardless of the type of crisis or ASD treatment received. The MLR analysis demonstrated that female patients who were exclusively on VPA monotherapy had higher mtDNA-CN than males. Therefore, VPA or VPA + LTG schemes differentially influenced this marker in female patients, so the function of mitochondrial response to this ASD therapy is unresolved.

The aging markers we have analyzed here are a measurement proxy of personal health outcomes and are easily damaged by ROS, systemic inflammation, and stress [66]. The TL and mtDNA-CN may be also coregulated via stress by hypothalamic–pituitary–adrenal axis activity, which in turn exacerbates the seizure occurrence, thus playing an important role in the development of epilepsy [67]. All the above features are present in patients with epilepsy and might be modulated by ASD therapy, but unfortunately, we were not able to evaluate them; therefore, further studies are warranted to address them.

Considering that the world population is aging, epilepsy in the elderly is an important and growing health problem. These elderly patients suffer from numerous clinically significant comorbidities; therefore, biomarkers of aging may also be useful to identify elderly epilepsy patients who are at risk of multimorbidity to efficiently classify them as biologically older (or younger) than their chronological age. This might help explain the variability in disease evolution or response to VPA and/or LTG between individuals of the same age and opens a window to investigate geroprotective interventions for epilepsy.

The limitations of the present study were a limited sample size, lack of clinical data in patients regarding the time of use of previous ASDs, duration of the illness, cotreatments and comorbidities, weight and other metabolic data, and lifestyle; the design was transversal, and peripheral tissue was explored for aging markers. However, regarding this last point, a group of researchers reported a correlation between the TL in brain tissue and peripheral tissues (e.g., blood, saliva, buccal) in living human subjects with intractable epilepsy [68], thus, posing the possibility that at least the leukocyte TL might provide insights into the brain TL in these patients with a neurological disorder. In addition, the measurement of peripheral mtDNA-CN has been associated with gene expression in other tissues, suggesting that blood-derived mtDNA-CN can reflect metabolic health across multiple tissues [65]. Although this study evaluated a relatively small sample size, the study design allowed for the comparative analysis of ASD monotherapy vs. combined therapy on aging markers in epilepsy patients. Also, our results suggest that the TL might predict the studied ASD treatment responsiveness in epilepsy patients.

## 5. Conclusions

Treatment with VPA in monotherapy or a combined scheme differentially influenced only the TL in our patients. These findings should be confirmed in future studies to clarify the impacts of ASDs on biological aging. This type of research will allow for the identification of new mechanisms of action of ASDs, reveal cellular alterations in epilepsy, and allow for the development of new therapeutic strategies. In addition, further longitudinal studies are required to achieve a full understanding of the role of ASD therapy (not only VPA and LTG) on different aging markers in patients with epilepsy.

## Figures and Tables

**Table 1:** Demographic and clinical characteristics of patients with epilepsy (*n* = 64) and healthy controls (*n* = 64).

Characteristics	Patients (*n* = 64)			Controls (*n* = 64)		
	Total (*n* = 64)	Male (*n* = 31)	Female (*n* = 33)	Total (*n* = 64)	Male (*n* = 31)	Female (*n* = 33)
Sex (%)	100	48	52	100	48	52
Age in years, mean ± SD (range)	32.0 ± 13.10 (18–73)	32.48 ± 14.20 (18–72)	31.6 ± 12.3 (18–73)	32.0 ± 13.0 (18–73)	32.5 ± 14.30 (73–18)	31.4 ± 11.9 (19–72)
Not seizure-free patients *	42	19	23	NA	NA	NA
Seizure-free patients *	22	12	10	NA	NA	NA
LTG monotherapy group	Patients (*n* = 18)			Controls (*n* = 18)		
	Total (*n* = 18)	Male (*n* = 7)	Female (*n* = 11)	Total (*n* = 18)	Male (*n* = 7)	Female (*n* = 11)
Sex (%)	100	39	61	100	39	61
Age in years, mean ± SD (range)	34.2 ± 14.9 (18–72)	39.6 ± 27.7 (18–72)	30.8 ± 9.39 (19–49)	34.3 ± 15.3 (18–73)	40.0 ± 21.4 (18–73)	30.6 ± 9.17 (19–47)
Not seizure-free patients *	13	5	8	NA	NA	NA
Seizure-free patients *	5	2	3	NA	NA	NA
LTG dose in mg; mean ± SD	225.0 ± 113.0	236.0 ± 103.0	218.0 ± 123.0	NA	NA	NA
LTG PC, *n* = (subtherapeutic/therapeutic/supratherapeutic)	(7/11/0)	(3/8/0)	(4/3/0)	NA	NA	NA
LTG PC μg mL^−1^; mean ± SD	4.6 ± 3.6	2.8 ± 1.6	5.74 ± 4.1	NA	NA	NA
LTG adjusted PC (µg mL^−1^ dose Kg^−1^)	1.5 ± 1.5	0.9 ± 0.3	2.0 ± 1.8	NA	NA	NA
VPA monotherapy group	Patients (*n* = 19)			Controls (*n* = 18)		
	Total (*n* = 19)	Male (*n* = 9)	Female (*n* = 10)	Total (*n* = 19)	Male (*n* = 9)	Female (*n* = 10)
Sex (%)	100	57	43	100	57	43
Age in years, mean ± SD (range)	32.8 ± 12.3 (20–67)	34.4 ± 14.8 (21–67)	31.0 ± 9.42 (20–49)	32.7 ± 12.0 (21–66)	34.2 ± 14.5 (21–66)	31.0 ± 9.19 (21–49)
Not seizure-free patients * (*n* = 9)	9	4	5	NA	NA	NA
Seizure-free patients * (*n* = 10)	10	5	5	NA	NA	NA
VPA dose in mg; mean ± SD ^⤉^	932.0 ± 437.0	1100.0 ± 477.0	844.0 ± 397.0	NA	NA	NA
VPA PC, *n* = (subtherapeutic/therapeutic/supratherapeutic) ^⤉^	(0/17/0)	(0/9/0)	(0/8/0)	NA	NA	NA
VPA PC, μg mL^−1^; mean ± SD ^⤉^	68.1 ± 20.6	60.3 ± 16.7	76.9 ± 22.0	NA	NA	NA
VPA adjusted PC (µg mL^−1^ dose Kg^−1^) ^⤉^	4.4 ± 1.3	4.0 ± 1.2	5.0 ± 1.2	NA	NA	NA
LTG + VPA combined therapy group	Patients (*n* = 27)			Controls (*n* = 27)		
	Total (*n* = 27)	Male (*n* = 14)	Female (*n* = 13)	Total (*n* = 27)	Male (*n* = 14)	Female (*n* = 13)
Sex (%)	100	52	48	100	52	48
Age in years, mean ± SD (range)	30.1 ± 12.6 (18–73)	27.6 ± 7.64 (19–48)	32.8 ± 16.4 (18–73)	29.9 ± 12.3 (18–72)	27.6 ± 7.44 (18–47)	32.4 ± 15.9 (19–72)
Not seizure-free patients *	20	9	11	NA	NA	NA
Seizure-free patients *	7	5	2	NA	NA	NA
LTG dose in mg; mean ± SD	189.0 ± 84.7	211.0 ± 92.4	165.0 ± 71.8	NA	NA	NA
LTG PC, *n* = (subtherapeutic/therapeutic/supratherapeutic)	(1/21/5)	(1/10/3)	(0/11/2)	NA	NA	NA
LTG PC, μg mL^−1^; mean ± SD	9.8 ± 4.3	9.8 ± 4.6	9.7 ± 4.0	NA	NA	NA
LTG adjusted PC (µg mL^−1^ dose Kg^−1^)	3.7 ± 1.8	3.2 ± 0.8	4.1 ± 2.4	NA	NA	NA
VPA dose in mg; mean ± SD	1039.0 ± 496.0	1189.0 ± 476.0	950 ± 122.0	NA	NA	NA
VPA PC, *n* = (subtherapeutic/therapeutic/supratherapeutic) ^§^	(0/15/1)	(0/6/0)	(0/9/1)	NA	NA	NA
VPA PC, μg mL^−1^; mean ± SD ^§^	74.8 ± 25.5	76.8 ± 15.9	80.0 ± 33.9	NA	NA	NA
VPA adjusted PC (µg mL^−1^ dose Kg^−1^) ^§^	2.7 ± 1.6	1.1 ± 1.5	2.1 ± 2.0	NA	NA	NA

* Seizure-free patients: according to the operational definition of the International League Against Epilepsy (ILAE), this is when patients have gone without a seizure for at least 3 times the duration of their longest pre-intervention inter-seizure interval in the preceding 12 months (www.ilae.org (accessed on 12 December 2024)). PC: plasma concentration; subtherapeutic PC of LTG (<3 µg mL^−1^); therapeutic PC of LTG (3–15 µg mL^−1^); supratherapeutic PC of LTG (>15 µg mL^−1^); subtherapeutic PC of VPA (<8.9 µg mL^−1^); therapeutic PC of VPA (8.9–115 µg mL^−1^); supratherapeutic PC of VPA (>115 µg mL^−1^); ^⤉^ data from 17 patients; ^§^ data from 16 patients; NA: not applicable.

## Data Availability

The data presented in this study are available upon request from the corresponding author. The data are not publicly available due to ethical issues.

## Supplementary Materials

The following supporting information can be downloaded at https://www.mdpi.com/article/10.3390/jcm14010255/s1: Table S1: Adverse drug reactions observed in patients with epilepsy treated with antiseizure drugs; Table S2: Summary of the correlation analysis between the types of adverse antiseizure drug reactions and the aging markers in epilepsy patients.
