# Supplementary material for: Valproic Acid and Lamotrigine Differentially Modulate the Telomere Length in Epilepsy Patients
Source: J Clin Med. 2025 Jan 3;14(1):255. doi: 10.3390/jcm14010255 (PMC11720991; doi:10.3390/jcm14010255)
Supplement: Supplementary file 1 [file jcm-14-00255-s001.zip › jcm-3394931-supplementary.pdf]

Table S1. Adverse drug reactions observed in patients with epilepsy treated with antiseizure drugs.

Adverse drug reactions (ADRs)	Total patients (%) (n=64)	Patients on LTG (%) (n=18)	Patients on VPA (%) (n=19)	Patients on LTG+VPA (%) (n=27)
**General**				
Nervousness or distress	38.9	25.0	5.9	69.2
Fatigue or tiredness	35.6	50.0	11.8	42.3
Drowsiness	35.6	18.8	41.2	42.3
Weight gain	39.0	31.3	47.1	38.5
Insomnia	32.2	43.8	11.8	38.5
Alopecia	25.4	18.8	11.8	38.5
Feeling groggy	22.0	12.5	5.9	38.5
Thick or swollen gums	20.7	31.3	11.8	20.0
Hyperactivity	13.6	6.3	5.9	23.1
Sexual dysfunction	10.2	12.5	5.9	11.5
Weight loss	8.6	12.5	5.9	8.0
Hirsutism	6.8	6.3	0	11.5
**Gastrointestinal**				
Abdominal pain or gastritis	33.9	18.8	11.8	57.7
Constipation	23.7	12.5	17.6	34.6
Diarrhea	13.6	6.3	17.6	15.4
Nausea and/or vomiting	13.6	0	11.8	23.1
**Cutaneous**				
Allergy (mild rash)	5.1	0	5.9	7.7
Allergy (moderate or severe rash or Steven Johnson)	0	0	0	0
Facial edema	0	0	0	0
Toxic epidermal necrolysis (Lyell syndrome)	0	0	0	0
**Neurological**				
Memory failure	50.8	43.8	35.3	65.4
Difficulty concentrating	45.8	31.3	35.3	61.5
Headache	42.4	56.3	17.6	50.0
Shaking (tremors)	35.6	18.8	29.4	50.0
Dizziness or vertigo	27.1	12.5	17.7	42.3
Trouble speaking	25.4	25.0	17.6	30.8
Slow thinking	23.7	25.0	11.8	30.8
Confusion	18.6	18.8	29.4	11.5

Double or blurred vision or nystagmus	16.9	18.8	5.9	23.1
Difficulty walking (ataxia)	13.6	12.5	11.8	15.4
Instability	10.2	0	11.8	15.4
Paresthesia	8.5	6.3	0	15.4
Parkinsonism	8.5	6	11.8	7.7
Diplopia	0	0	0	0
Choreoathetosis	0	0	0	0
**Psychiatric**				
Aggression or irritability	50.8	50	29.4	65.4
Depression or sadness	47.5	43.8	47.1	50.0
Humor changes	37.3	31.3	52.9	30.8
Hallucinations, agitation, delirium	13.6	6.3	5.9	23.1
Manic episode	6.8	0	5.9	11.5
Suicidal ideation	6.8	6.3	17.6	0

Table S2. Summary of the correlation analysis between types of antiseizures adverse drug reactions and the aging markers in epilepsy patients.

Aging markers	Antiseizure therapy				
**LTG monotherapy group (p-value / r-value)**					
	**General**	**Gastrointestinal**	**Cutaneous**	**Neurological**	**Psychiatric**
**TL**	0.086 / 0.218	0.740 / -0.042	0.971 / -0.005	0.414 / -0.105	0.268 / -0.142
**mtDNA-CN**	0.198 / 0.164	0.554 / -0.076	0.540 / -0.079	0.514 / 0.084	0.401 / -0.108
**VPA monotherapy group (p-value / r-value)**					
	**General**	**Gastrointestinal**	**Cutaneous**	**Neurological**	**Psychiatric**
**TL**	0.429 / -0.193	0.362 / 0.222	0.196 / 0.310	0.459/ -0.1809	0.802 / 0.062
**mtDNA-CN**	0.359 / -0.223	0.594 / 0.131	0.330 / -0.237	0.746 / 0.070	0.889 / -0.035
**LTG+VPA combination therapy group (p-value / r-value)**					
	**General**	**Gastrointestinal**	**Cutaneous**	**Neurological**	**Psychiatric**
**TL**	0.290 / 0.216	0.068 / -0.364	0.890 / 0.029	0.253/ -0.232	0.121 / -0.311
**mtDNA-CN**	0.130 / 0.305	0.128 / -0.306	0.782 / -0.057	0.777 / 0.058	0.341 / -0.195
